# Adaptive cranial simulation programme: matching procedural challenge and simulation capability to learner need

**DOI:** 10.3389/fsurg.2026.1977123

**Published:** 2026-10-27

**Authors:** Adam F. Roche, Dara O. Kavanagh, Darach Crimmins, Lena-Mary Houlihan, Gulam Zilani, Vincent Healy, Jack Horan, Claire M. Condron

**Affiliations:** 1 RCSI SIM Centre for Simulation Education and Research, RCSI University of Medicine and Health Sciences, Dublin, Ireland; 2 Department of Surgical Affairs, RCSI University of Medicine and Health Sciences, Dublin, Ireland; 3 Department of Neurosurgery, Temple Street Hospital, Dublin, Ireland; 4 School of Medicine, University College Dublin, Dublin, Ireland; 5 Department of Neurosurgery, Cork University Hospital, Cork, Ireland; 6 Department of Neurosurgery, Beaumont Hospital, Dublin, Ireland

**Keywords:** adaptive learning, curriculum design, neurosurgical education, simulation-based education (SBE), technical skills

## Abstract

**Introduction:**

Procedural simulation must provide meaningful learning for trainees with differing levels of operative experience. We developed a mixed-level cranial simulation programme designed to align procedural challenge, available simulation resources and faculty support with individual learner needs.

**Curriculum design and delivery:**

A half-day cranial simulation programme was delivered twice annually, combining parallel foundational and advanced circuits, with procedural content informed by a previously published needs assessment. Activities were allocated according to operative experience, observed progression and anticipated clinical responsibilities rather than training grade alone. Readiness-based stretch tasks provided selected trainees with supervised exposure to more advanced procedural challenges.

**Implementation considerations:**

Simulation modality, instrumentation and supporting technology were matched to procedural complexity and learning objectives. Authentic operative equipment and advanced simulation technologies were incorporated where appropriate, with industry partnerships supporting access to specialist resources. The parallel circuit structure enabled foundational and advanced activities to be delivered concurrently while concentrating specialist faculty, equipment and higher-capability simulation resources where they were most relevant to learner needs.

**Lessons learned and next steps:**

This report describes curriculum design and implementation rather than effectiveness. Future development will extend selected stations from isolated technical skills towards scaffolded rehearsal of complete procedural workflows and integrate structured formative assessment throughout practice. A stable foundational curriculum with adaptable advanced content may provide a practical framework for supporting learners with differing levels of experience with a shared simulation programme.

## Introduction

Simulation-based education (SBE) can support safe development of procedural skills that may be difficult to acquire through clinical exposure alone (1). Procedural SBE can be delivered using a range of modalities, such as simple task trainers, low-cost synthetic or anatomical models, cadaveric training and technology-enhanced platforms (2). The appropriate modality depends on the intended learning objectives, procedural task, learner experience and available resources. Some advanced procedural activities may require additional capability, including specialist equipment, authentic instrumentation, advanced or complex simulators, simulation technical expertise (3), faculty time or suitable teaching facilities. The challenge is therefore not that procedural simulation inherently depends on sophisticated infrastructure, but that available educational resources should be selected and deployed where they best support clinically relevant learning at an appropriate level of challenge.

We have encountered this challenge in several ways. SBE time and resources may be dedicated to standardised procedural training because a skill forms part of a generic curriculum or minimum competency requirement, despite limited relevance to a trainee's anticipated clinical responsibilities. This can result in learners practicing procedures that they are unlikely to perform in the near term, while those approaching more complex operative responsibilities may lack access to SBE of sufficient procedural or technological capability. Conversely, sophisticated simulators and equipment do not necessarily optimise learning if they are used for activities poorly matched to the learner's current needs. These mismatches represent potentially low-value use of limited training time and resources that could otherwise be directed towards more clinically relevant learning. The challenge is therefore not simply to provide more SBE or greater fidelity, but to direct limited SBE time, faculty expertise and technology towards clinically relevant procedural practice at an appropriate level of challenge. Appropriate SBE fidelity, task complexity and instructional support should be aligned with the learning objective, the learner's existing experience and the clinical context in which transfer is expected (4).

In smaller surgical specialties, relatively limited trainee numbers and specialist faculty can make separate SBE programmes for different stages of training impractical, while repeated course delivery may also increase demands on clinical service provision. Bringing trainees across the training pathway together within a single SBE programme provides a pragmatic means of concentrating faculty, equipment and trainee time within fewer educational events, but creates the challenge of meeting substantially different learning needs simultaneously. However, a mixed-experience cohort may also provide opportunities for peer learning and readiness-based progression, allowing selected trainees to observe or engage with more advanced procedural practice when appropriate.

In our surgical skills laboratory, trainees from surgical training (ST) years 2–8 therefore participate in a half-day cranial SBE programme delivered twice annually. Rather than providing identical activities according to training grade, we developed parallel foundational and advanced circuits within a shared curriculum architecture. Individual activities were adapted in real time according to operative experience, observed progression and anticipated clinical responsibilities. This approach allows shared SBE infrastructure to support learners across a broad spectrum of experience while concentrating specialist faculty, technology and resources within a single educational event.

The contribution of this curriculum lies not in the introduction of new educational principles, but in their deliberate integration and practical application within a mixed-experience neurosurgical SBE programme. Established principles, including progressive complexity, scaffolding, readiness-based progression and formative feedback, are operationalised through a shared curriculum model in which procedural challenge, SBE capability and faculty support are adapted to individual learner need.

This article describes the design and implementation of this curriculum, including the principles used to allocate procedural challenge, integrate SBE technology and specialist equipment, support readiness-based progression and structure faculty coaching. We also describe practical lessons from implementation, including the planned integration of structured formative assessment, limitations of the approach and priorities for future evaluation.

### Curriculum design and delivery

The curriculum was designed not simply to separate trainees into foundational and advanced activities, but to align each learner's next clinically relevant procedural challenge with an appropriate level of complexity, the SBE capability required to support it, and the faculty coaching and feedback needed to support progression. These elements formed part of a broader set of educational design principles used to guide curriculum implementation across the mixed-experience cohort (Table 1). Procedural content was selected using an evidence-informed approach, reflecting established principles of systematic curriculum development (5). Procedural content was selected using an evidence-informed approach, reflecting established principles of systematic curriculum development (5). Procedural content was drawn from a previously published Delphi-based general needs assessment that identified and prioritised procedures and approaches considered important for neurosurgical SBE (6). The needs assessment identified training priorities across the neurosurgical curriculum but did not prescribe their allocation according to training stage. Within the present curriculum, consultant faculty applied a targeted needs assessment approach, interpreting these priorities according to procedural complexity, expected operative exposure and anticipated clinical responsibilities to determine their appropriate placement within the foundational or advanced circuit. Individual task allocation was subsequently adapted according to each trainee's experience and observed progression rather than training grade alone. This provided a stable curriculum core while allowing individual activities to vary in complexity. The foundational circuit remained relatively constant, reflecting core cranial skills required throughout training, whereas the advanced circuit retained greater flexibility to respond to evolving clinical practice and training needs. For example, neuroendoscopy could be supplemented or replaced by neuro-oncology SBE as priorities change, without altering the overall curriculum architecture.

**Table 1:** Educational design principles underpinning the adaptive cranial simulation curriculum. Summary of the curriculum's educational design principles, their implementation and intended educational purpose.

Educational design principle	Curriculum implementation	Educational purpose
Progressive procedural complexity	Foundational and advanced circuits provide procedural activities of increasing complexity, with opportunities for readiness-based progression	Match procedural challenge to the learner's developmental stage
Adaptive task allocation	Faculty consider previous operative exposure, anticipated next clinical responsibilities and observed performance during the session to select and adapt procedural activities in real time	Direct each learner towards the most clinically relevant task at an appropriate level of challenge
Purpose-matched simulation capability	Simulation modality, instrumentation and supporting technology are matched to the requirements of the selected procedure and learner	Ensure the simulation environment supports the intended level of procedural practice without unnecessary use of resource-intensive technology
Readiness-based progression	Learners demonstrating appropriate foundational performance may undertake a supervised stretch task when educationally appropriate and logistically feasible	Provide exposure to the next level of procedural complexity without implying independent competence
Shared simulation infrastructure	Stations, equipment and faculty support multiple levels of procedural complexity within the same training event	Concentrate limited faculty, equipment and simulation resources while supporting different learner needs
Stable but responsive curriculum architecture	The foundational circuit remains relatively stable, while advanced procedural themes can evolve with clinical practice, technology and training priorities	Maintain a consistent curriculum framework while preserving relevance to contemporary clinical practice and training needs

The national neurosurgical training cohort comprised 19 trainees across ST2 to ST8 at the time of implementation, including 10 trainees at ST2 to ST4 and nine at ST5 to ST8. Cohort size varied between years according to the number of allocated training posts. The half-day programme was delivered twice annually, with trainees typically attending one of the two sessions depending on clinical service requirements, although some were able to attend both. Each delivery commenced with brief faculty-led didactic teaching addressing relevant procedural anatomy, surgical approaches, equipment and technical considerations before trainees progressed to hands-on SBE. Although the curriculum was not explicitly designed around Kolb's experiential learning cycle, its delivery incorporated features consistent with experiential learning, with preparatory teaching followed by hands-on procedural experience, faculty-guided reflection and feedback, and opportunities to modify and repeat technical performance during subsequent practice (7). Foundational and advanced circuits were then delivered concurrently, comprising four stations each and supported by eight consultant faculty, with one faculty member allocated to each station (Figures 1, 2). Stations generally accommodated two trainees, who alternated between primary operator and assistant roles while receiving direct coaching. The foundational circuit primarily accommodated ST2 to ST4 trainees, while ST5 to ST8 trainees participated in the advanced circuit, although individual task allocation and readiness-based progression were not determined by training grade alone. At the start of the practical course component, faculty and trainees agreed task plans based on previous operative exposure and anticipated clinical responsibilities, with further adaptation occurring in real time according to observed progression. Within a shared procedural theme, complexity could vary between learners. For example, cranial access ranged from decompressive hemicraniectomy to bifrontal or posterior fossa decompression, while the burr-hole station supported EVD insertion or chronic subdural haematoma evacuation incorporating operative planning and surface anatomy discussion. For EVD insertion, a purpose-designed cranial simulator provided realistic tissue feel during catheter passage, tactile feedback on ventricular entry and simulated cerebrospinal fluid return (Figure 3). This enabled the same SBE infrastructure to provide different levels of technical and cognitive challenge according to learner need.

**Figure 1:**
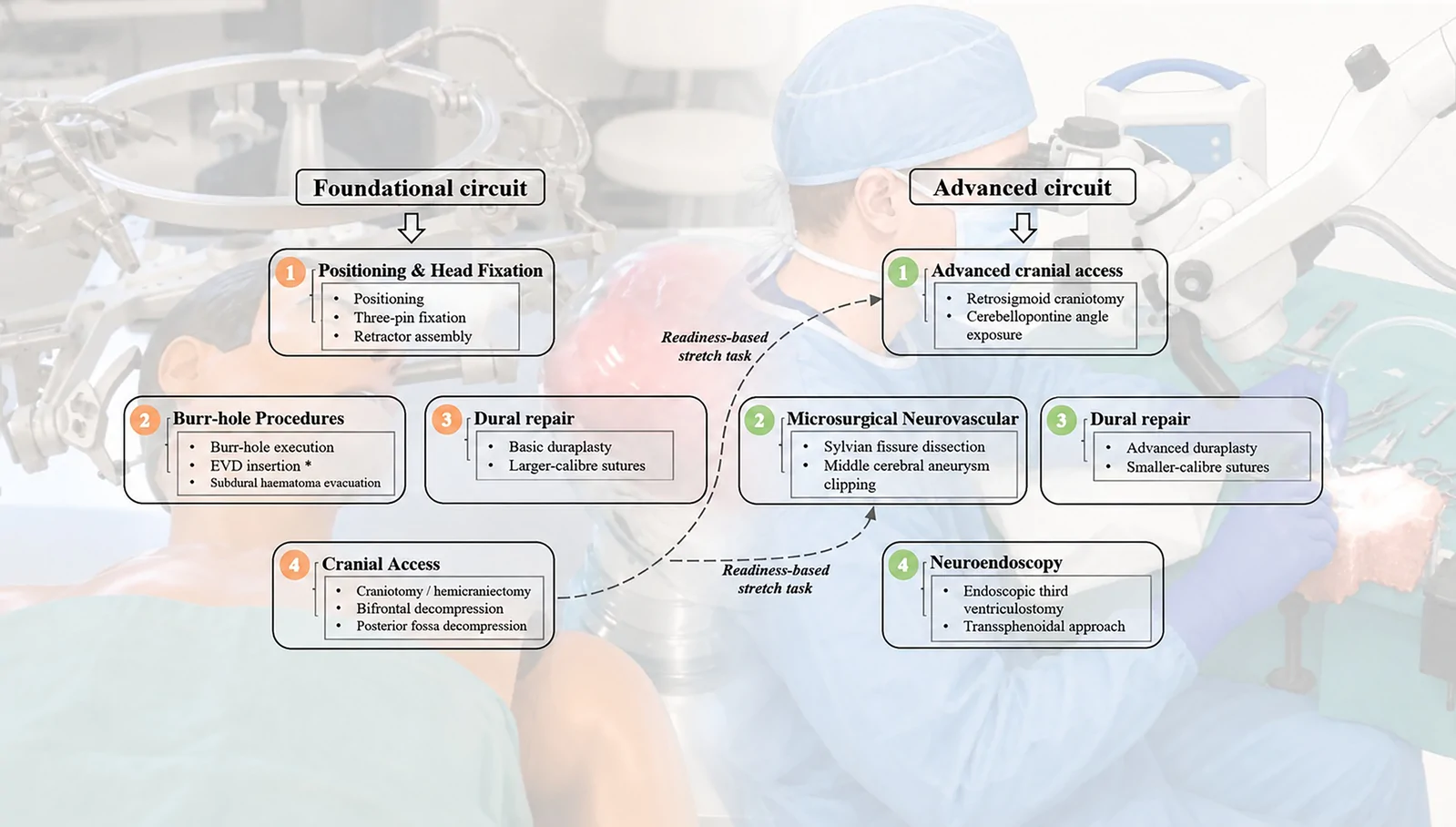
Adaptive cranial SBE curriculum for mixed-experience neurosurgical trainees. Foundational and advanced circuits provide procedural activities of differing complexity within a shared SBE programme. Individual activities are adapted according to operative experience, observed progression and anticipated clinical responsibilities rather than training grade alone. Readiness-based stretch tasks provide selected trainees with supervised exposure to advanced procedural challenges beyond their usual circuit. * EVD - external ventricular drain.

**Figure 2:**
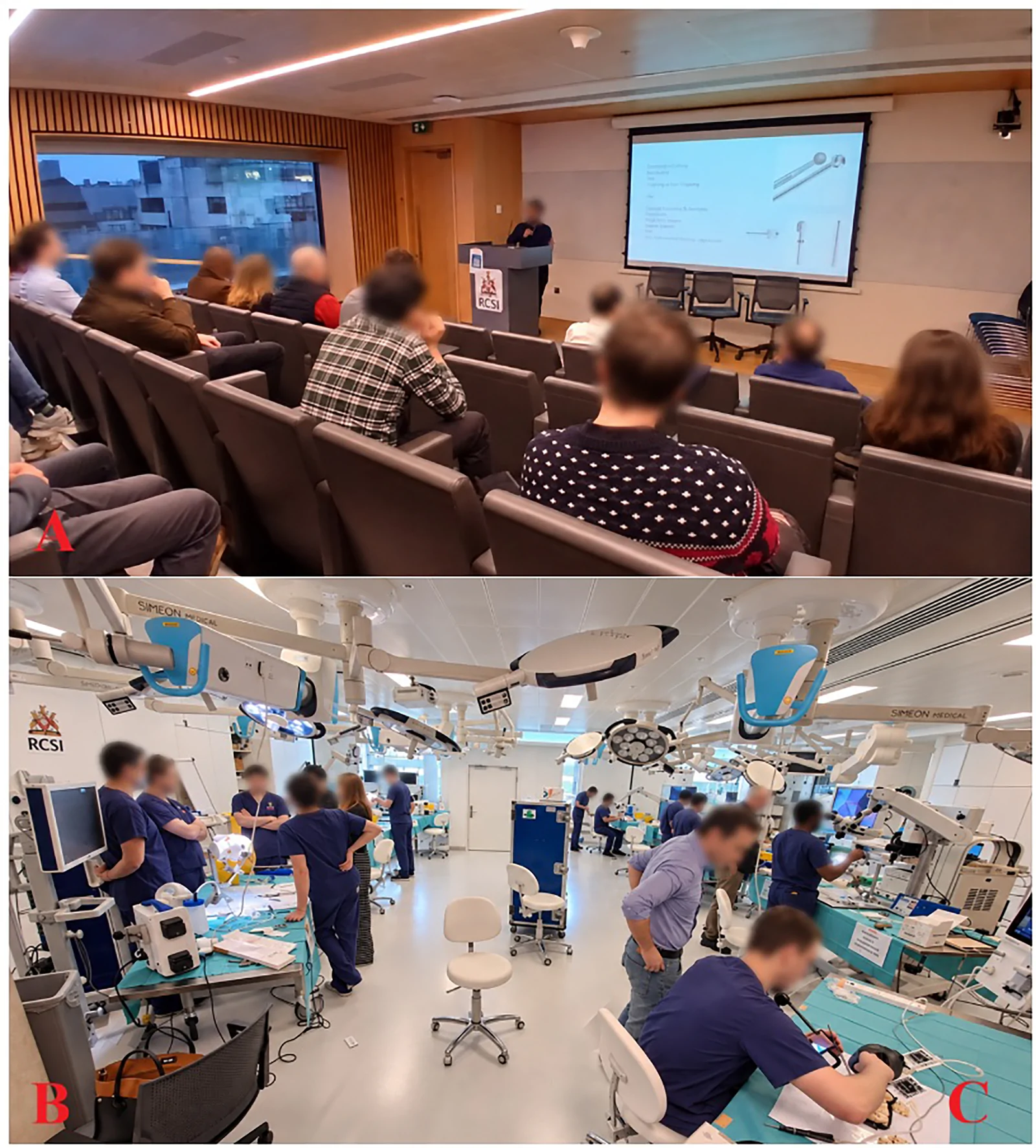
Delivery of the mixed-experience cranial SBE programme. **(A)** Faculty-led didactic teaching before practical simulation, addressing relevant procedural anatomy, surgical approaches, equipment and technical considerations. **(B)** Foundational technical-skills circuit and **(C)** advanced technical-skills circuit delivered concurrently within the shared surgical skills laboratory, with trainees undertaking supervised procedural practice and receiving direct faculty coaching and formative feedback.

**Figure 3:**
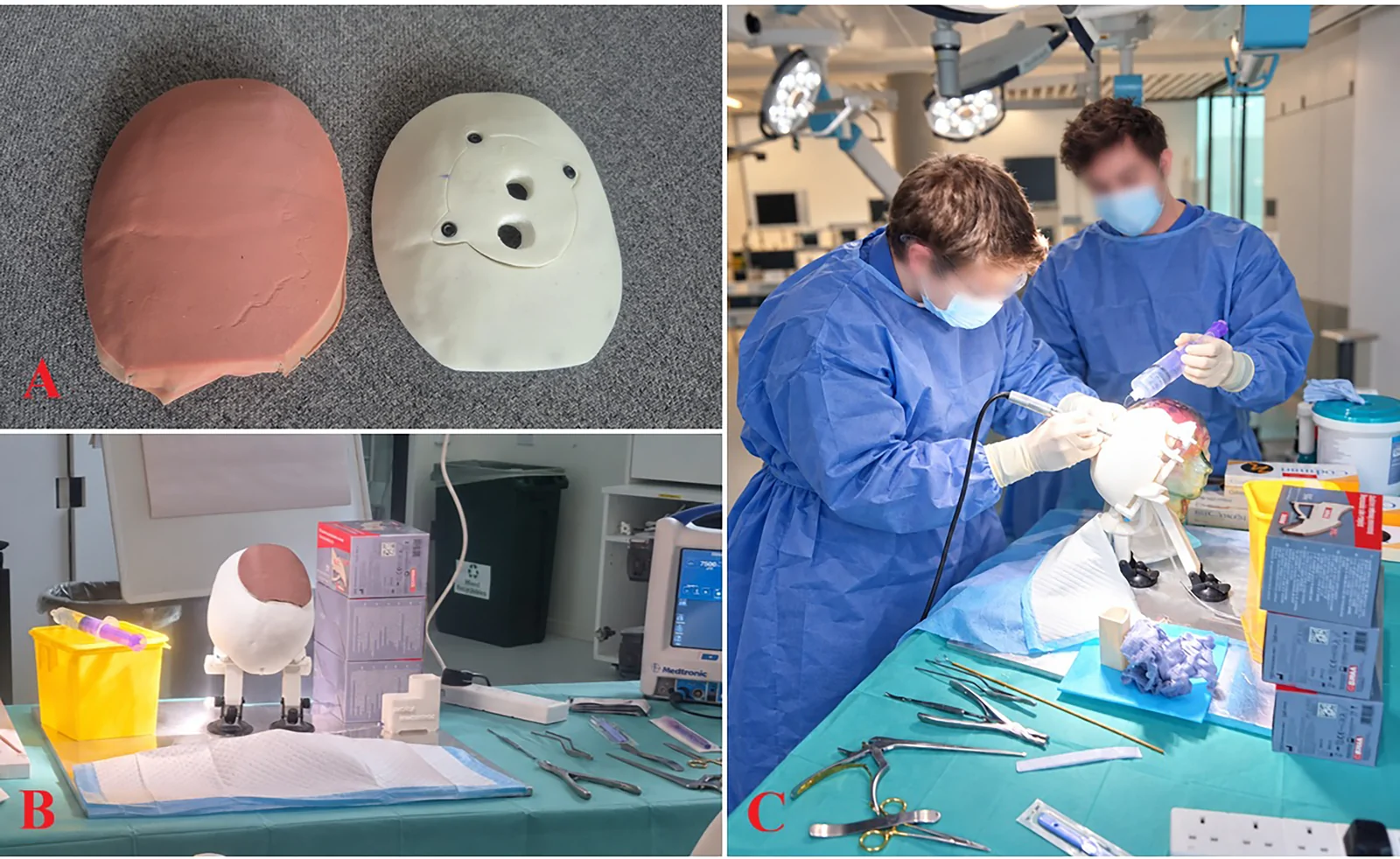
External ventricular drain (EVD) insertion simulation model and procedural practice. **(A)** Cranial simulator components, demonstrating the scalp and skull components used for procedural access. **(B)** EVD simulation station configured for procedural practice, with the assembled cranial simulator, relevant instrumentation and supporting equipment. **(C)** Trainee performing simulated EVD insertion with an assistant. The simulator provides realistic tissue feel during catheter insertion, tactile feedback on ventricular entry and simulated cerebrospinal fluid return. The EVD simulator is the subject of a pending patent application by Tactile Innovations Ltd.

Selected trainees (typically ST4) could also be offered a readiness-based stretch task, comprising supervised participation in one advanced-circuit station beyond their usual pathway (Figure 1). Stretch tasks were optional rather than a required stage of progression and were offered only when the trainee demonstrated sufficient confidence and familiarity with the foundational activities and was judged by consultant faculty to be ready for additional challenge. Stretch tasks were preferentially selected from advanced stations representing a logical progression of skills already encountered within the foundational circuit. Advanced cranial access extended established skills in positioning, operative planning, drilling and craniotomy into more anatomically constrained approaches, such as retrosigmoid craniotomy with cerebellopontine angle exposure. Microsurgical neurovascular tasks extended foundational tissue handling, dural work and anatomical orientation through microscope-based dissection, Sylvian fissure opening and neurovascular exposure. These stations therefore enabled trainees to integrate and extend established component skills within more complex procedural tasks, rather than introducing an unrelated advanced procedure solely on the basis of training grade. Readiness was determined through faculty judgement of previous operative exposure, performance and procedural understanding demonstrated during the foundational circuit, and the relevance of the advanced task to anticipated clinical responsibilities. This judgement was not intended to represent a validated assessment of competence.

### Implementation considerations

Matching procedural complexity to learner experience also required the SBE environment to support the intended level of practice. In keeping with the principle of purpose-matched SBE capability (Table 1), authentic clinical equipment was incorporated where it directly supported the learning objective, including head-fixation and retractor systems, operating microscopes, microsurgical instrumentation, neuroendoscopy and surgical navigation. Simulator capability, instrumentation and supporting technology were scaled to the requirements of each activity. For example, the transsphenoidal skills station used a previously validated, anatomically detailed sinonasal and skull-base model incorporating relevant nasal, paranasal sinus, pituitary and neurovascular anatomy (8). This enabled trainees to develop three-dimensional anatomical understanding while practising endoscopic access, drilling and microdebrider use. Corresponding CT datasets supplied with full-head simulators could be registered to surgical-navigation systems, enabling image-guided procedural practice (Figure 4).

**Figure 4:**
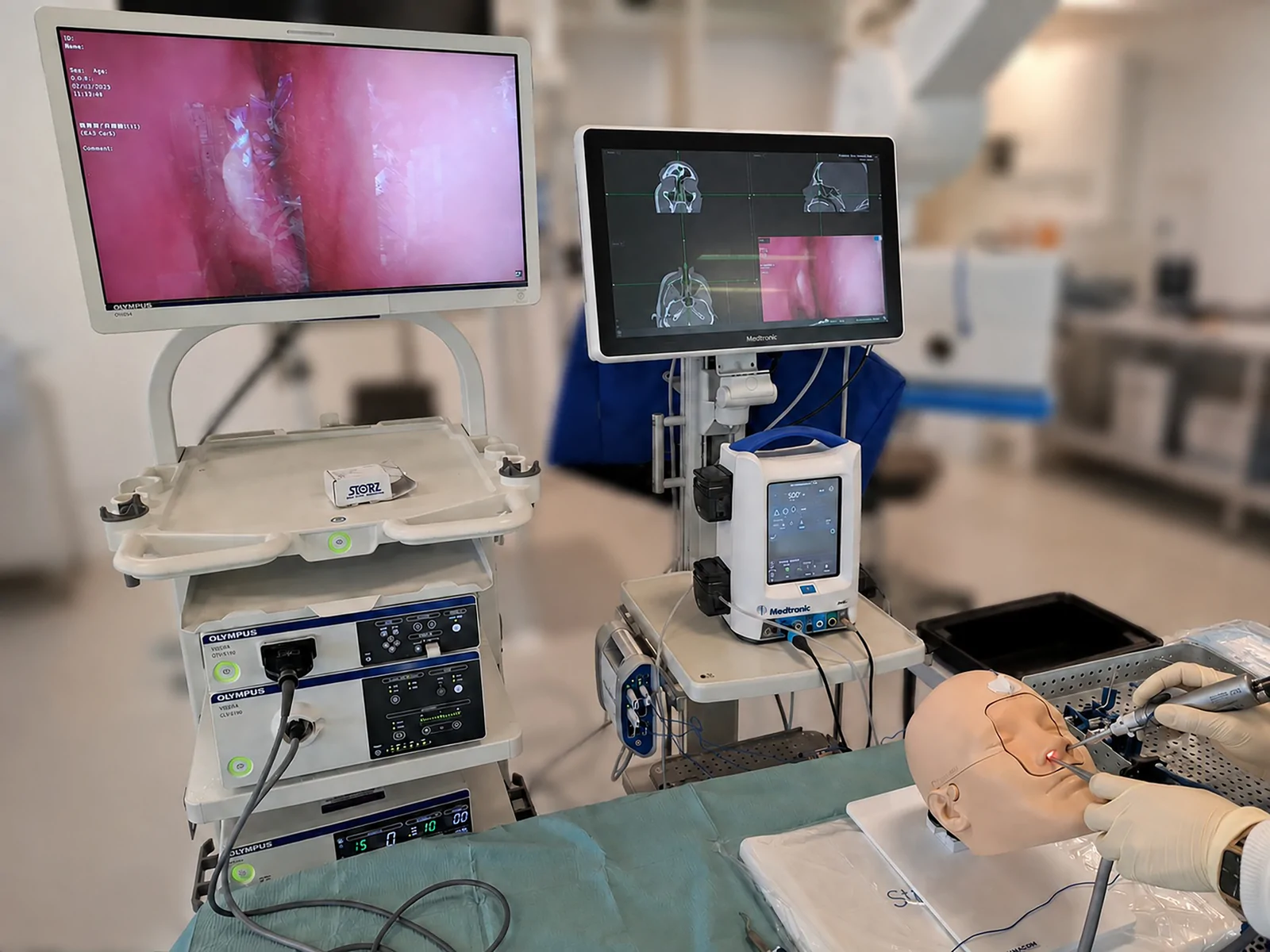
Purpose-matched SBE capability for advanced neuroendoscopic training. The station integrates a CT-compatible full-head simulator with endoscopic imaging, surgical navigation, functional endoscopic sinus surgery instrumentation and relevant neurosurgical instruments. This configuration supports rehearsal of image-guided anatomical orientation, endoscopic access and transsphenoidal skull-base approaches. The station illustrates how simulator capability, instrumentation and supporting technology can be aligned with procedural demands and the anticipated clinical responsibilities of advanced trainees.

Providing this level of procedural fidelity required considerable preparation and access to specialist equipment, some of which was required for only part of the course. Established relationships with industry partners enabled selected technologies and instrumentation to be borrowed where permanent ownership was neither practical nor necessary. Beyond improving access to resource-intensive equipment, these collaborations enabled trainees to practice using authentic operative instruments and technologies encountered in clinical care. Familiarity with the operation, handling and integration of these systems may reduce the gap between SBE and clinical practice and support subsequent transfer of learning. Importantly, high-fidelity technology was not considered an educational objective in itself; resources were selected according to the requirements of the procedure, learning objectives and learner needs. The underlying curriculum model therefore does not depend on reproducing the same facilities, equipment or technologies at every institution. Advanced technologies were used in this programme only when they provided capability required by the intended learning objective, such as image guidance, endoscopic access or microscope-based microsurgical practice. Where these technologies are unavailable, activities can be adapted to locally available modalities while preserving alignment between clinical relevance, learner need and procedural complexity. The transferable element is therefore the educational allocation principle of matching the available modality, procedural challenge and faculty support to individual learner need, rather than any requirement to reproduce a technology-rich SBE environment. More broadly, meaningful procedural SBE does not require access to a dedicated SBE centre or sophisticated technology. Procedural skills can be practised using a spectrum of locally available modalities, from simple, cost-effective or locally developed models (9) to cadaveric and more technologically advanced approaches, according to the intended learning objectives. The relevant consideration is whether the available modality can support clinically relevant practice at an appropriate level of challenge.

### Lessons learned and next steps

Implementation highlighted that isolated technical-skills practice remains valuable for developing foundational competence but that, where feasible, learners may benefit from subsequently integrating these skills within the wider procedural workflow. We are therefore extending selected stations using a scaffolded approach, in which learners first develop foundational component skills before progressively integrating them within the complete procedure (10). For EVD insertion, this means progressing beyond isolated catheter placement to rehearsal of the complete technical procedural sequence. In related work currently under peer review, a consensus-derived EVD workflow comprising 22 steps was developed, with steps classified as essential, context-dependant or variable. Rehearsing this sequence enables trainees to practice not only catheter insertion but also the complete sequence of associated technical actions, with the aim of reinforcing procedural sequencing and recognition of critical steps before clinical practice.

We also recognised that adaptive task allocation should be accompanied by equally structured feedback. Although not yet implemented within the curriculum, future iterations of the curriculum will integrate formative assessment throughout procedural practice using defined domains from established technical-skills assessment frameworks, such as the Objective Structured Assessment of Technical Skills (OSATS) (11). For EVD insertion, in response to an identified clinical need to reinforce consistent procedural practice, we plan to complement this global assessment with a task-specific checklist derived from the 22-step consensus workflow described above. The checklist will support structured assessment of procedural step completion, including recognition of essential procedural actions, while OSATS will provide a broader assessment of technical performance. The workflow steps are currently classified as essential, context-dependent or variable rather than numerically weighted, and the combined approach will initially be used formatively rather than to establish a pass/fail or competency threshold. A global framework such as OSATS can provide a broad indication of technical performance across different procedural activities while supporting structured feedback. For other procedures within the curriculum, developing and validating separate task-specific assessment instruments for each activity would be resource intensive and is not required for the intended formative purpose. Rather than using assessment primarily to determine competence, faculty can use these domains to identify technical strengths, target areas for improvement and agree subsequent learning goals while simulated practice is ongoing. Introducing structured formative assessment may also strengthen learner engagement and motivation by providing greater purpose, accountability and a clearer sense of progression during each task.

Finally, the curriculum should remain responsive without requiring continual redesign. The foundational circuit provides a relatively stable core of essential cranial skills, whereas advanced stations can evolve as clinical practice, technology and training priorities change. This permits new procedural themes to be introduced within the existing architecture while retaining the mixed-level, readiness-based approach. Although developed for neurosurgery, these principles may be applicable to surgical specialties in which learners at different stages train together using shared faculty, equipment and SBE resources. The approach aims to increase the educational value of resource-intensive SBE by aligning clinically relevant procedural challenges, SBE capability and faculty support with each learner's developmental needs.

### Evaluation and limitations

This report describes curriculum design and implementation rather than an effectiveness study. The programme has been delivered twice annually over several years, with trainees attending one or both half-day deliveries each year according to clinical and training requirements. However, the present report does not include longitudinal or comparative data to establish the durability of learning, the effect of repeated participation across training, technical performance improvement, transfer to clinical practice or patient level outcomes.

The curriculum was implemented within a relatively small national neurosurgical training programme in which faculty were familiar with many trainees’ operative experience. Its feasibility and scalability in larger cohorts, institutions with different faculty to trainee ratios, or settings with less established knowledge of individual trainees remain to be evaluated.

Future evaluation should examine implementation feasibility across repeated deliveries; trainee perceptions of relevance, challenge and psychological safety; faculty acceptability; task allocation decisions; uptake of readiness based stretch tasks; and structured formative assessment data. Longitudinal evaluation should assess retention of technical performance, the contribution of repeated participation across the training pathway and, where feasible, indicators of transfer to supervised clinical practice.

## Conclusion

This adaptive curriculum provides a practical approach to delivering clinically relevant SBE across a mixed-experience surgical cohort within shared faculty, infrastructure and time constraints. By aligning procedural challenge, SBE capability and faculty support with individual learner needs and anticipated clinical responsibilities, the approach aims to maximise the educational value of resource-intensive SBE while remaining responsive to evolving training needs.

## Data Availability

The original contributions presented in the study are included in the article/Supplementary Material, further inquiries can be directed to the corresponding author.
